# Partition Function and Configurational Entropy in Non-Equilibrium States: A New Theoretical Model

**DOI:** 10.3390/e20040218

**Published:** 2018-03-23

**Authors:** Akira Takada, Reinhard Conradt, Pascal Richet

**Affiliations:** 1 Innovative Technology Research Center, Asahi Glass Co. Ltd., Yokohama 221-8755, Japan; 2 Department of Earth Sciences, University College London, London WC1E 6BT, UK; 3 Glass and Composites and Institute of Mineral Engineering, RWTH Aachen University, 52064 Aachen, Germany; 4 Institut de Physique du Globe de Paris, 1 Rue Jussieu, 75005 Paris, France

**Keywords:** configurational entropy, thermodynamics, statistical mechanics, computer simulation

## Abstract

A new model of non-equilibrium thermodynamic states has been investigated on the basis of the fact that all thermodynamic variables can be derived from partition functions. We have thus attempted to define partition functions for non-equilibrium conditions by introducing the concept of pseudo-temperature distributions. These pseudo-temperatures are configurational in origin and distinct from kinetic (phonon) temperatures because they refer to the particular fragments of the system with specific energies. This definition allows thermodynamic states to be described either for equilibrium or non-equilibrium conditions. In addition; a new formulation of an extended canonical partition function; internal energy and entropy are derived from this new temperature definition. With this new model; computational experiments are performed on simple non-interacting systems to investigate cooling and two distinct relaxational effects in terms of the time profiles of the partition function; internal energy and configurational entropy.

## 1. Introduction

The glassy state and the glass transition constitute one of the long-standing research fields that focus on non-equilibrium states [1,2]. In these cases, a popular phenomenological approach is based on de Donder’s affinity concept [3]. For the Helmholtz representation, it is expressed as:

Δ*F* = Δ*U* − *T*Δ*S*: in equilibrium, (1)

Δ*F* = Δ*U* − *T*Δ*S* + *A*(*T*)*dξ*: in non-equilibrium, (2)

where *F*, *U*, *T* and *S* are the Helmholtz free energy, internal energy, temperature and entropy, respectively. As for the internal thermodynamic parameter *ξ*, usually termed advancement of reaction, it reflects the degree of structural deviation from the equilibrium state whereas the affinity *A* indicates the rate at which the system relaxes toward its equilibrium state. Regarding glass, the usefulness of this phenomenological approach has been demonstrated by the application of a hole-lattice model that yielded the entropy, entropy production and glass transition temperature as a function of the cooling and heating rates of the system [4].

For glasses, another fruitful approach [5] relies on the distinction made between the actual temperature of the system, as defined by that of the heat bath in contact with it, and its fictive temperature, i.e., the temperature at which equilibrium has been lost upon cooling and the atomic configuration has been frozen in. At equilibrium, the fictive and actual temperatures are by definition identical. In contrast, the former remains constant whereas the latter continuously decreases when the glass is cooled below the glass transition. In view of its simplicity and operational validity this concept is commonly used in industry to evaluate the effects of thermal history on glass properties [6].

The common point between the de Donder formulation and the fictive temperature approach is that only one order parameter (*ξ* or fictive temperature) specifies a given non-equilibrium state. In spite of their practical interest, however, such models with one order parameter have limitations that have called for more complex analyses [7]. In this respect, our new approach is to make use of the concept of spatial sampling of microstates, whose advantages over ensemble and temporal samplings have been pointed out in our previous discussions of entropy in non-equilibrium systems [8,9,10]. Rather than modifying Equation (1) or (2), we begin with the construction of an extended canonical partition function. We then calculate relevant thermodynamic variables on the basis of this fundamental concept of statistical mechanics. Finally, we report the results on computational experiments performed on simple non-interacting systems to investigate cooling and relaxation effects in terms of the time profile of the partition function, internal energy and configurational entropy.

## 2. Theoretical Model Description

The simplest way to relate a new non-equilibrium model to the well-established equilibrium thermodynamic theory is to consider non-interacting particles so that one can arrive at an analytical formulation. To simplify further the problem, we will consider only the configurational part of the partition function.

Our starting point is the traditional canonical ensemble for a system composed of *N* particles. For convenience, the energy *e*(*i*) of particle *i* is referred to a scale whose zero is the lowest particle energy in the system. The single-particle partition function per particle then is defined as:

(3) $$z_{c}=\sum \exp[-e\left(i\right)/(k_{B}T)]$$

where *k_B_* and *T* are Boltzmann constant and the system temperature. The parameter *i* is varied over *all states* that a particle *may* occupy, which is based on ensemble sampling. The partition function obtained in Equation (3) then is rewritten in another way:

(4) $$z_{c}=\sum g_{c}\left(j\right)\exp[-e\left(j\right)/(k_{B}T)]$$

where *g_c_*(*j*) is the number of degenerate states of energy *e*(*j*). The parameter *j* is thus varied over *all energy levels*.

The configuration integral for the whole system comprised of *N* particles is:

(5) $$Z_{c}=z_{c}^{N}.$$

Once *z_c_* or *Z_c_* is calculated, the Helmholtz free energy *A_c_*, internal energy *E_c_*, and configurational entropy *S_c_* are easily calculated from Equation (3) as follows:

(6) $$A_{c}/N=-k_{B}Tln\left(z_{c}\right)$$

(7) $$E_{c}/N=\sum e\left(i\right)[\exp\left(-e\left(i\right)/\left(k_{B}T\right)\right)/z_{c}]$$

(8) $$S_{c}/N=\left(\frac{E_{c}}{N}-\frac{A_{c}}{N}\right)/T=\left(E_{c}/N\right)/T+k_{B}\ln\left(z_{c}\right)$$

Alternatively, we will now make use of the concept of “spatial sampling” whereby particles are chosen randomly in the whole three-dimensional space. The partition function then is approximated by:

(9) $$z_{s}=d\;\sum \exp[-e\left(k\right)/(k_{B}T)],$$

where *d* and *e*(*k*) are a scaling factor and the energy sampled from the sample labelled *k*, respectively. The application of “spatial sampling” is discussed in more detail in Appendix A. Since the parameter *k* is varied over *all samplings*, a more accurate information will be obtained with a higher number of samplings. In contrast, *z_s_* should not depend on this number of samplings. Hence, the problem is to evaluate the *d* parameter each time the total number of samplings is changed because the total number of states is not usually known a priori. Since our main concern is to calculate non-equilibrium thermodynamic properties, a simple way to solve indirectly this difficulty is to estimate the *d* parameter such that the results obtained via either the ensemble or spatial sampling are in mutual agreement for equilibrium states. In this study, since the analytical form (Equation (17)) is known, the *d* parameter can be easily estimated. Non-equilibrium calculations with the obtained *d* parameter can then be performed as explained in the remaining part of this section. An investigation of more general cases and another way to estimate directly the *d* parameter will be discussed in a future study.

Turning now to the extension of traditional statistical mechanics to non-equilibrium states, we first note that the canonical partition function is defined for a single temperature so that it leads to only one Boltzmann factor-like energy distribution. Since any arbitrary energy distribution can occur in non-equilibrium, this restriction must be removed. For this purpose, we introduce the concept of *pseudo-temperature* to characterize a given energy level at a microscopic scale, with which an extended canonical partition function is defined as:

(10) $$z_{n}=d\sum \exp[-e\left(j\right)/(k_{B}T\left(j\right))],$$

where *T*(*j*) designates a function of the energy level *e*(*j*). The *j* parameter is summed over *all sampling* such that *T*(*j*) can have a different value for each sampled fragment. For estimating *T*(*j*) the procedure is first to perform the spatial sampling, identify its energy and then to calculate *z_n_*. In the same way as the *d* parameter is estimated for equilibrium states, each *T*(*j*) is calculated term by term to match the sampled distribution. It follows that Equation (10) can be converted into:

(11) $$z_{n}=\sum \exp[-e\left(i\right)/(k_{B}T\left(i\right))],$$

with the difference that Equation (10) is summed over *all sampling* whereas Equation (11) is in contrast summed over *all states*. The concept of *pseudo-temperature* is discussed in more detail in Appendix B.

Next, the internal energy is calculated. The probability of the state labelled *i* in Equation (11) is:

(12) $$p\left(e\left(i\right)\right)=\exp\left[-e\left(i\right)/(k_{B}T\left(i\right)\right)]/z_{n}.$$

The expected configurational part of the energy per particle in the extended canonical ensemble is:

(13) $$e=\sum e\left(i\right)p\left(e\left(i\right)\right)=\sum e\left(i\right)\exp[-e\left(i\right)/(k_{B}T\left(i\right))]/z_{N}.$$

To calculate the configurational entropy, *s*, per particle, we use the Gibbs entropy formula:

(14) $$s=-k_{B}\sum \{p(e\left(i\right)\ln\left[p\left(e\left(i\right)\right)\right]\}$$

Substituting Equations (12) and (13) into Equation (14), we obtain:

(15) $$\begin{array}{c} S=-k_{B}\sum \{\exp[-e\left(i\right)/(k_{B}T\left(i\right))]/z_{n}[-e\left(i\right)/\left(k_{B}T\left(i\right)\right)-\ln\left(z_{n}\right)]\}\\ =k_{B}\sum [e\left(i\right)/(k_{B}T\left(i\right))\exp\left[-e\left(i\right)/\left(k_{B}T\left(i\right)\right)/z_{n}\right]\\ +k_{B}\sum \{\exp[-e\left(i\right)/(k_{B}T\left(i\right))]/z_{n}\}\ln\left(z_{n}\right)\\ =\sum \left[e\left(i\right)/\left(T\left(i\right)\right)p\left(e\left(i\right)\right)\right]+k_{B}\ln\left(z_{n}\right) \end{array}$$

Equations (13) and (15) are thus the desired extended versions of Equations (7) and (8).

## 3. Computational Model

We will use the cell theory for an application to some simple non-interacting systems of the partition function and configurational entropy derived in the previous section. This theory is popular to simulate crystals and liquids [11,12] because it is not only easily modeled, but also lends itself to analytical formulations for the traditional single-particle partition functions. A form of the model similar to that of a classical Einstein crystal is used here. Each particle moves in a spherically symmetric harmonic potential, *u* = (1/2)*k_c_r*^2^, where *k_c_* and *r* are the force constant and distance from the equilibrium position. With a simple Lennard-Jones-type potential the experimental thermodynamic properties in liquid states of Ar have been reasonably well reproduced [13]. In our study, we used consistently the parameters fitted to the liquid state of Ar at 161.73 K [13].

The Lennard-Jones potential is:

(16) $$u=4\varepsilon [{\left(\sigma /r\right)}^{12}-(\sigma /r{)}^{6}].$$

The parameters of *ε*/*k_B_* and *σ* were 119.8 K and 3.405 Å. The force constant *k_c_* was calculated from *k_c_σ*^2^/*ε* = 63.4. The fixed density *ρ* was calculated from *ρσ*^3^ = 0.80. The state referring to these conditions will be called here the standard case. Since a partial analytical integration is possible for this model, such an integration will be used instead of random sampling. Specifically, we sampled 1000 points between 0 and 5 Å at equal intervals along the distance axis. All reported results will be expressed in Å^3^ units for partition functions, *K* for internal energies, and *k_B_* for configurational entropies. As for the simulated time profiles, arbitrary time unit will be used. As shown in next section, MD calculations have not been performed in this study. Rather, we have made numerical integrations by using the analytical formula to calculate partition function. Our main objective is not to reproduce the results of [13] at equilibrium, but to design new theoretical model applicable to non-equilibrium states. It is in a future study that we will directly use the MD results carried out at non-equilibrium to construct a non-equilibrium partition function.

## 4. Results for Constant Temperatures

### 4.1. Standard Case in Equilibrium States

The single-particle partition function *z_r_* is expressed as the integration of the components of *p_r_*(*r*):

(17) $$\begin{array}{c} p_{r}\left(r\right)=\int_{0}^{\pi }\int_{0}^{2\pi }\exp[-e\left(r\right)/\left(k_{B}T\right)]r^{2}\sin\theta d\varphi d\theta \\ =[\exp(-e\left(r\right)/\left(k_{B}T))\right]\left[\int_{0}^{\pi }\int_{0}^{2\pi }r^{2}\sin\theta d\varphi d\theta \right]\\ =4\pi r^{2}\left[\exp\left(-e\left(r\right)/\left(k_{B}T\right)\right)\right] \end{array}$$

(18) $$z_{r}=\int_{0}^{\infty }p_{r}\left(r\right)dr.$$

Since the integrand of *p_r_*(*r*) is separated into the two terms shown in Equation (17), *z_r_* is also expressed as follows:

(19) $$z_{r}=\int_{0}^{\infty }g_{e}\left(r\right)g_{r}\left(r\right)dr=\int_{0}^{\infty }g\left(r\right)dr.$$

The term *g_e_*(*r*) depends on energy *e*(*r*) and temperature *T*. In contrast, the term *g_r_*(*r*) depends on the volume in which the particle with energy *e*(*r*) can move. They will be named the Boltzmann factor and the effective volume, respectively. Once a pair of *e*(*r*) and *g*(*r*) values is calculated, these can be converted as a function of energy as follows:

(20) $$z_{r}=\int_{0}^{\infty }g_{e}^{\prime }\left(e\right)g_{r}^{\prime }\left(e\right)de=\int_{0}^{\infty }g^{\prime }\left(e\right)de.$$

As a matter of fact this formulation makes it easier to consider the general situation where the center positions that define the *r* axis cannot be identified beforehand because of the random nature of the configurations.

**Figure 1:**
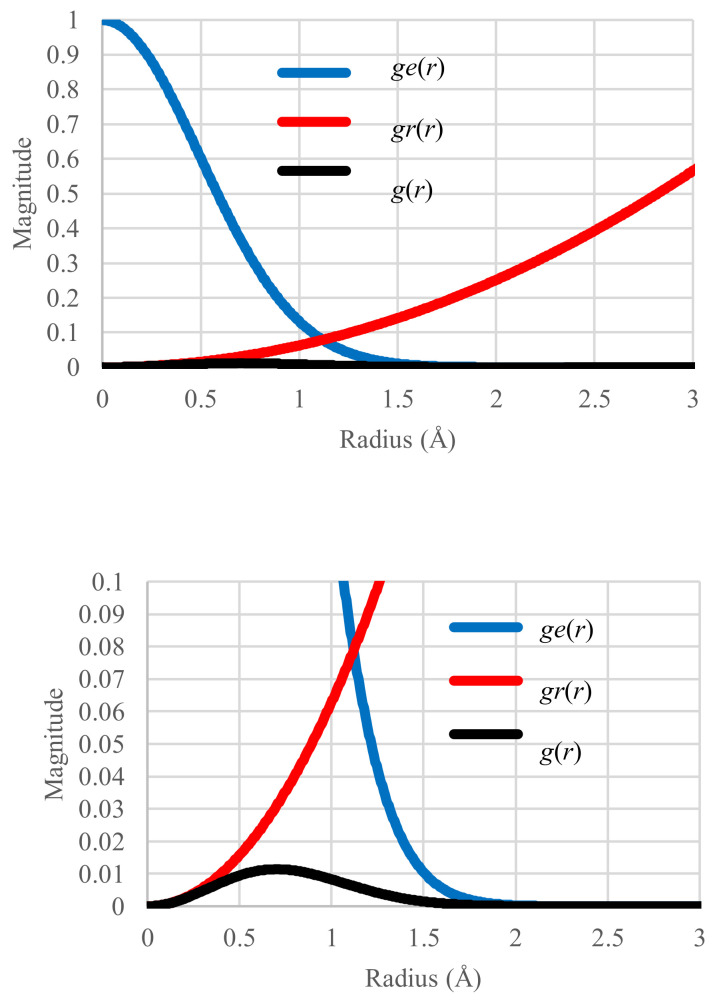
Distribution of Boltzmann factor *g_e_*(*r*), effective volume *g_r_*(*r*) and single partition function *g*(*r*) as a function of distance r in the standard case. Units of vertical axis: Å^2^. Small bump of the *g_r_*(*r*) distribution made visible in the lower, magnified plot.

We first plot the *g_e_*(*r*), *g_r_*(*r*) and *g*(*r*) distributions in the standard case (*T* = 161.73 K) in Figure 1 where the profile of *g*(*r*) represents the superposition of *g_e_*(*r*) and *g_r_*(*r*). The configurational energy and entropy were also calculated with Equations (13) and (15). Their values are 0.00790 K and 0.66779 *k_B_*, respectively.

### 4.2. Temperature Sensitivity in Equilibrium States

**Figure 2:**
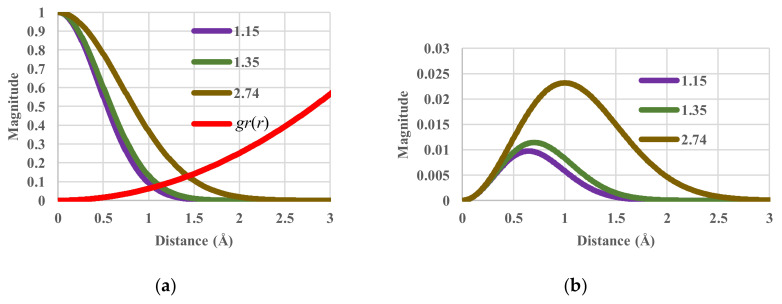
Partition functions against distance for values of *k_B_T*/*ε* of 1.15, 1.35 and 2.74. (**a**) Components of single-particle partition functions; (**b**) Single-particle partition function.

To illustrate the effects of temperature, the components of the single-particle partition functions and configurational entropy (*S_c_*/*N*) are shown in Figure 2. When the temperature (in units of *k_B_T*/*ε*) varies from 1.15 and 1.35 to 2.74, the corresponding value of the configurational energy varies from 0.00790 and 0.00927 to 0.01883 K. The corresponding values of the configurational entropy are 0.42591, 0.66779 and 1.73915 *k_B_*. Obviously, when temperature increases, entropy also increases as it should.

### 4.3. Effective-Volume Sensitivity in Equilibrium States

**Figure 3:**
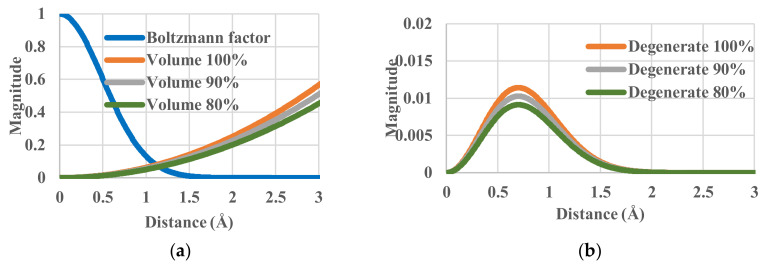
Partition functions against distance for relative effective volumes of 100%, 90% and 80%. (**a**) Components of single-particle partition functions; (**b**) Single-particle partition function.

To check the effects of effective volumes, we have calculated with Equation (17) the partition function through a spherical integration. By multiplying the *g_r_*(*r*) or *g_r_*’(*e*) terms and some constant value, we can artificially increase or decrease the effective volume in which the particles move under the same temperature conditions. This operation simulates some sort of blocking or pinning of particles. The effective volume was decreased from 100% (the standard case) to 90% and 80%. The components of single-particle partition function are shown in Figure 3. Although the Boltzmann factor *g_e_*(*r*) is invariant, the effective volume does vary. The corresponding values of entropy are 0.66779, 0.56243 and 0.44465 *k_B_*. All the corresponding configurational energies are 0.00927 K. There is no change in the effective energy balance between the system and the heat bath, i.e., no change in *g_e_*(*r*) in Equation (19), but the change of *g_r_*(*r*) reflects that of the partition function *z_r_* and consequently its entropy decreases.

## 5. Results for Constant Cooling Rates

### 5.1. Without Relaxation Effects

**Figure 4:**
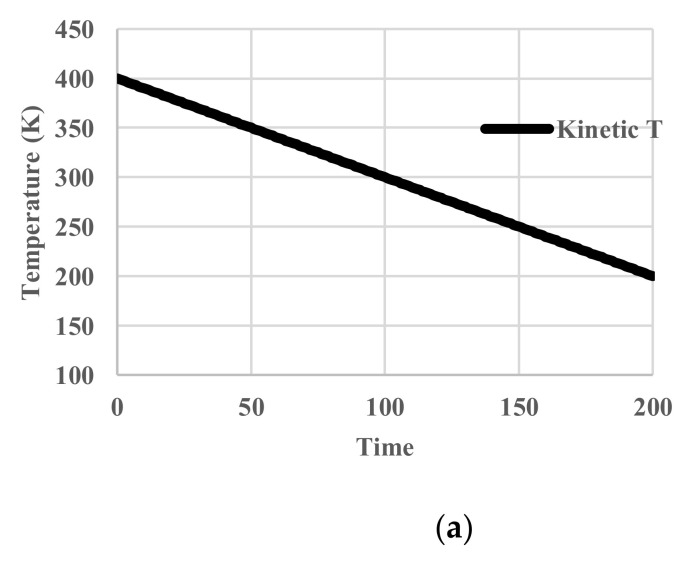

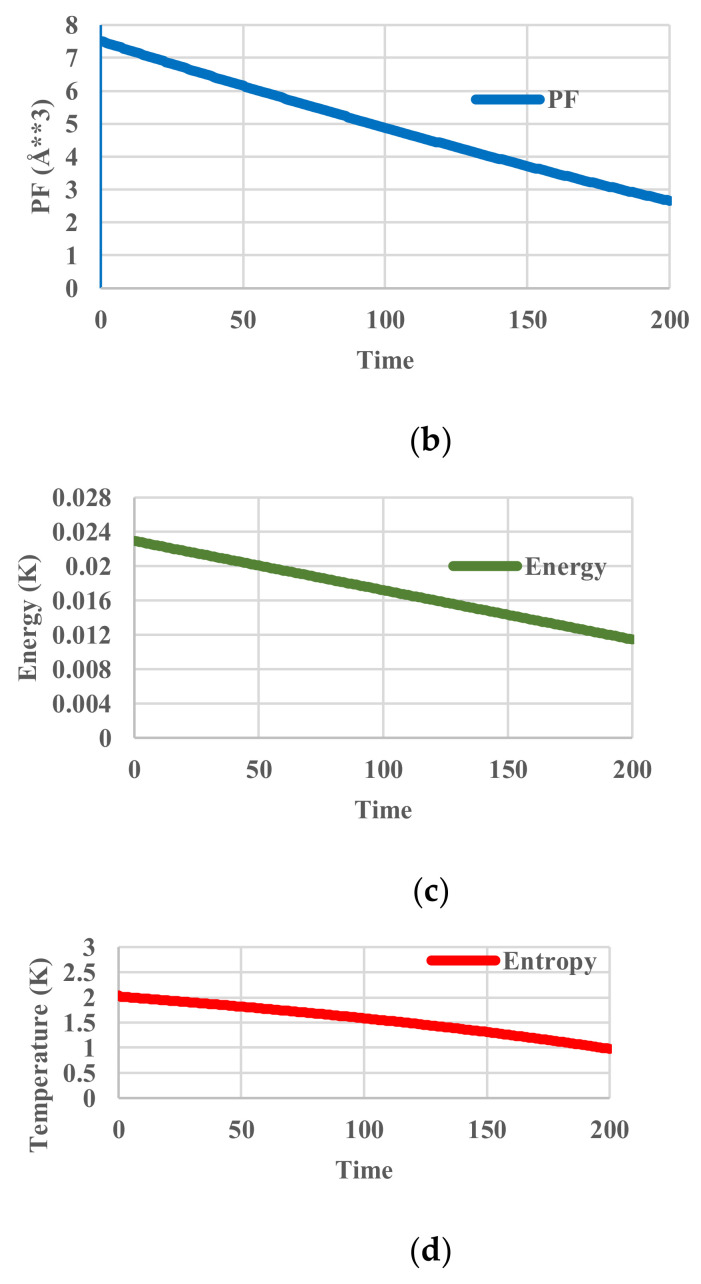
Cooling calculations without relaxation. (**a**) Profile of system (kinetic) temperature; (**b**) Profile of partition function *z_r_*; (**c**) Profile of configurational energy; (**d**) Profile of configurational entropy.

In this case, the temperature of the heat bath decreased from 400 K to 200 K. Since no relaxation effects are included, the temperature of the particles follows that of the heat bath. We name it “kinetic temperature” because the velocity distribution of the particles also adjusts to it instantaneously. This temperature is distinguished from the “configurational temperature”, because the latter reflects the potential energy distribution of the particles. The deviation of the latter from the former originates from relaxation effects. No unit of time is assigned. The profiles of given kinetic temperature, calculated partition function, configurational energy, and configurational entropy are shown in Figure 4. Obviously, all the thermodynamic variables decrease monotonously.

### 5.2. Arrhenius-Type Relaxation Effects

It is difficult to calculate relaxation effects caused by particle interactions in the present cell model. Therefore, a phenomenological relaxation model is introduced instead here. The simplest such model is written as:

(21) X(t)=Xeq−(Xeq−X(t))exp(−t/τ),

where *X*(*t*), *Xeq* and *τ* are some state variable at time *t*, its equilibrium value and the relaxation time, respectively. There are several ways to choose to which state variable this relaxation model is applied. Since the partition function is the key variable connecting the particle states and thermodynamic properties in the present study, Equation (21) was applied to the components of the partition function *z_r_*(*r*):

(22) $$z_{r}\left(r,t+\Delta t\right)=z_{r}\left(r,t\right)+\left(z_{r}^{e}\left(T_{k}\right)-z_{r}\left(r,t\right)\right)\Delta t/\tau \left(T_{k}\right),$$

where *T_k_* and *z_r_^e^*(*T_k_*) is the kinetic (or heat bath) temperature and the equilibrium value of *z_r_^e^* at this temperature. Although our theoretical framework focuses on the relaxation of the partition function, no special properties are actually concerned as they would also relax in the same manner. With respect to the free energy, for example, the partition function nonetheless differs by the fact that it can be defined with a non-equilibrium heat bath. Its relaxation then originates either in the temperature dependence of the Boltzmann factor or in changes in the effective volume.

The relaxation time is assumed to follow an Arrhenian law so as to increase as the temperature decreases:

(23) $$\tau \left(T_{k}\right)=A+B/T_{k},$$

where *A* and *B* are constants. Historically, the temperature-dependency of melt viscosity was found to be well approximated with the form of Equation (23) in which *A* and *B* are fitting parameters. Without any special physical meaning, the values of *A* and *B* have been set to be 1 and 4000 for simplicity.

Relaxation phenomena involve non-equilibrium states. As explained above, each component of *z_r_*(*x*,*t*) has its own pseudo-temperature, which will now be termed “configurational temperature”. The component of the partition function, *p_r_*(*x*,*t*) is expressed in a general form as *exp*(−*e*(*r*)/*k_B_T*(*r*,*t*)). At equilibrium, *T*(*r*,*t*) is equal to the temperature of the heat bath or “kinetic temperature”. As back calculated from *p_r_*(*x*,*t*) under non-equilibrium conditions, *T*(*r*,*t*) in contrast differs from the “kinetic” temperature of the heat bath to become instead the “configurational temperature”. The configurational temperature calculated for each sampled fragment denotes the temperature in which the fragment holds the same probability of appearance as in equilibrium. Therefore, the *T*(*r*,*t*) varies from place to place depending on the deviation from “kinetic temperature”.

**Figure 5:**
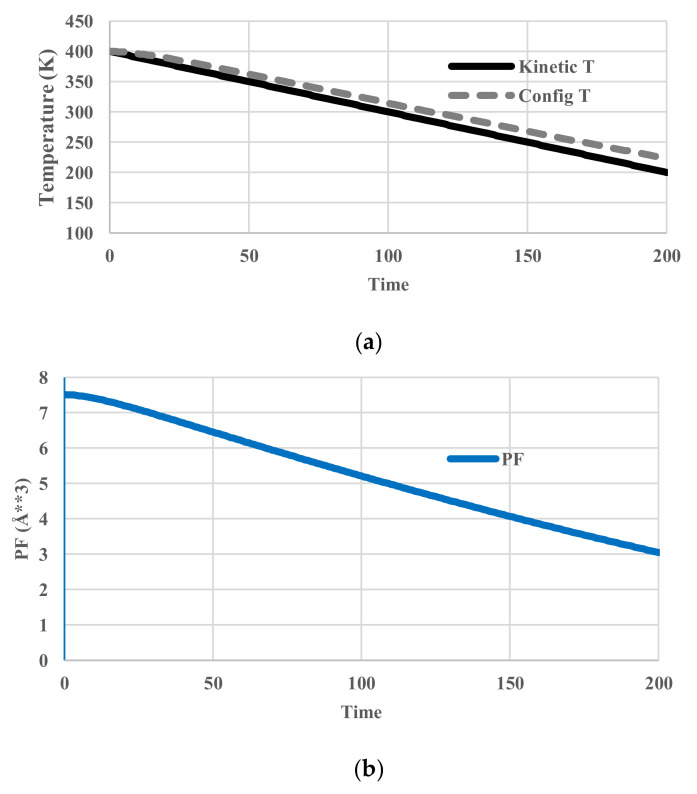

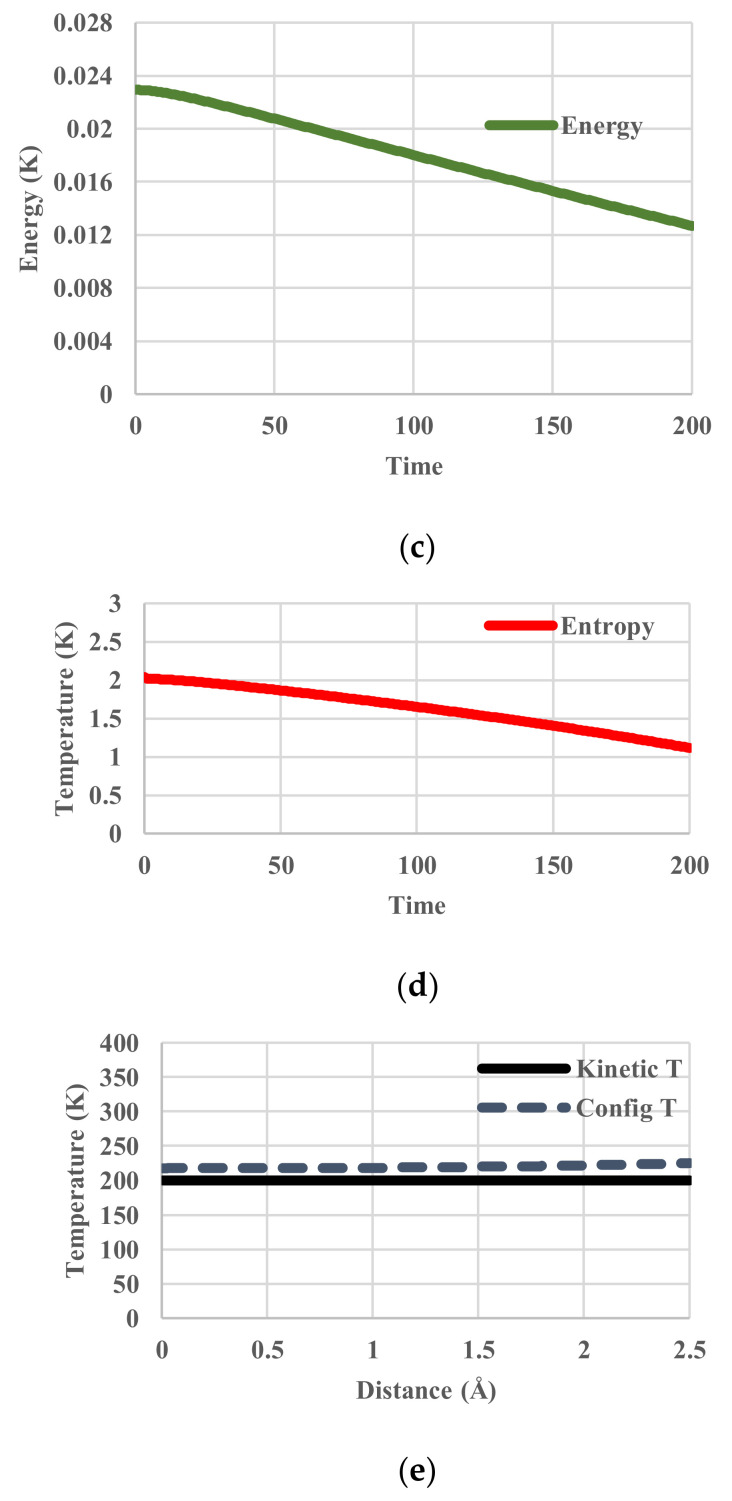
Cooling calculations with an Arrhenius-type relaxation. (**a**) Profile of system (kinetic) and averaged configurational temperature; (**b**) Profile of partition function; (**c**) Profile of configurational energy; (**d**) Profile of configurational entropy; (**e**) Spatial distribution of kinetic and configurational temperature.

The calculated temperatures, partition functions, configurational energies, and configurational entropies are shown in Figure 5. The average configurational temperature deviates more from the kinetic temperature as the system (kinetic) temperature decreases (Figure 5a). The configurational temperature (Figure 5a) is averaged over the spatial distribution of *T*(*j*) in Equation (11). As shown in Figure 5e, another interesting feature is that the distribution of configurational temperature is not flat.

### 5.3. Vogel-Fulcher-Tammann-Type Relaxation Effects

The next modification is to include a glass-like transition. Phenomenologically, the Vogel-Fulcher-Tammann (VFT) viscosity model is frequently used. For the relaxation time, it assumes:

(24) $$\tau \left(T_{k}\right)=A+B/\left(T_{k}-C\right),$$

where *A*, *B*, *C* are constants and the time constant diverges when the temperature approaches *C*. Without any special physical meaning, the values of *A*, *B* and *C* have been set to 1, 4000 and 251 for simplicity.

**Figure 6:**
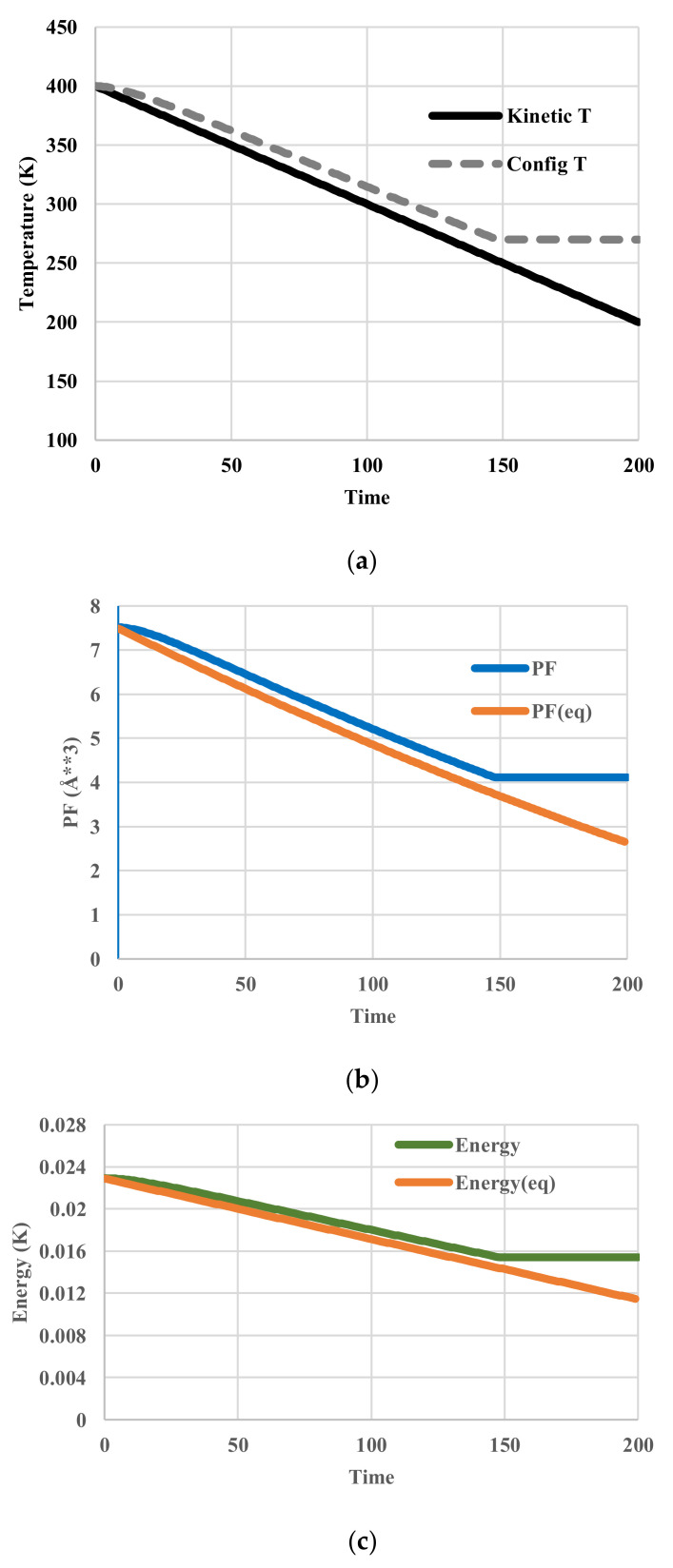

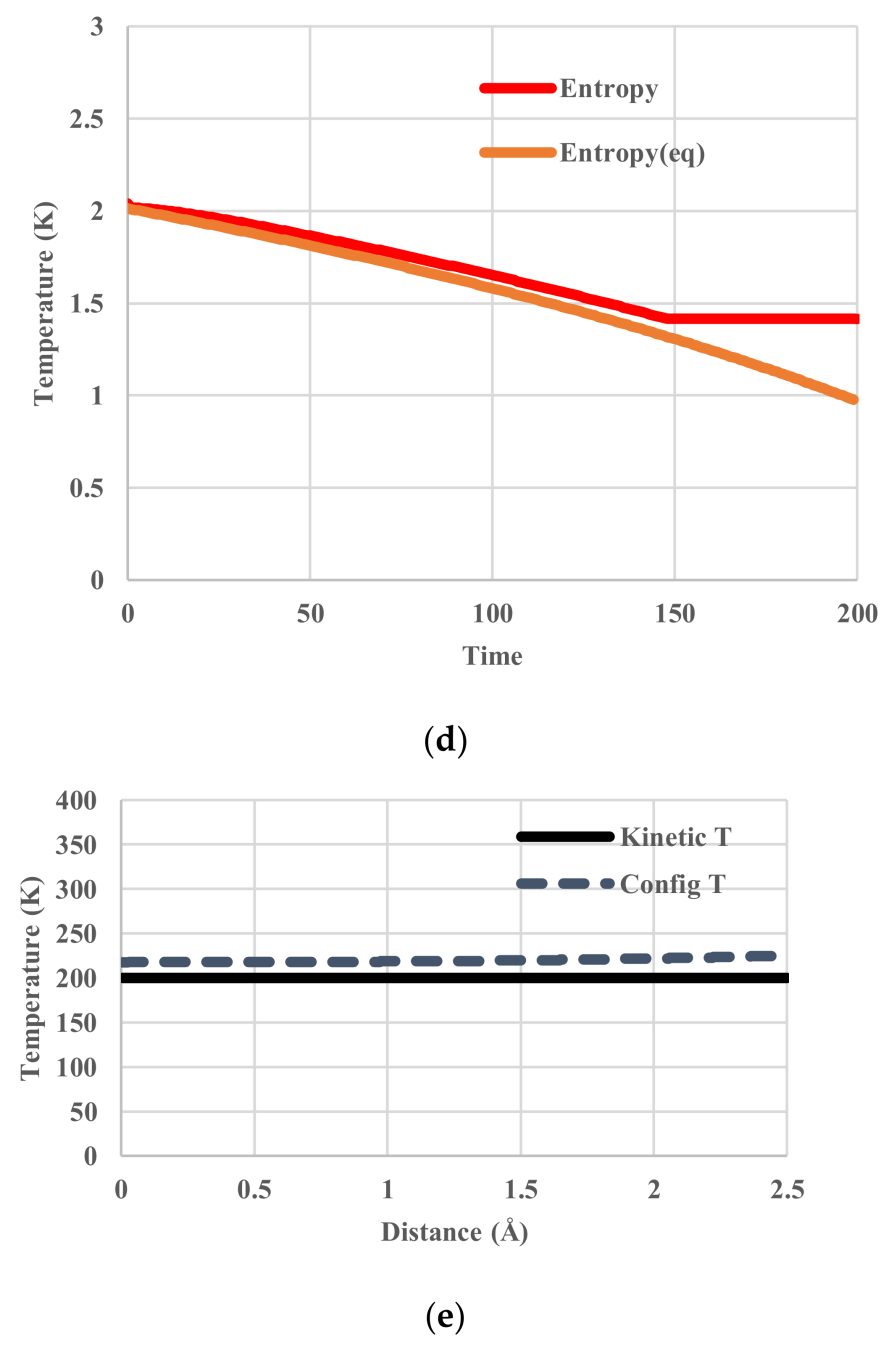
Cooling calculations with a VFT-type relaxation. (**a**) Profile of kinetic and spatially configurational temperature; (**b**) Profile of partition function; (**c**) Profile of configurational energy; (**d**) Profile of configurational entropy; (**e**) Spatial distribution of kinetic and configurational temperature.

The calculated temperatures, partition functions, configurational energies, and configurational entropies are shown in Figure 6. To figure out the deviations from those in equilibrium, the corresponding equilibrium values are also plotted in Figure 6b–d. At equilibrium, configurational temperatures in Figure 6a,e merge with kinetic temperatures. In contrast to those represented in Figure 5a,d, all these thermodynamic variables clearly exhibit a glass transition around the temperature of 251 K at which the configuration of the system has been frozen in.

## 6. Discussion

Two important factors in the partition function will first be discussed because they are key issues to understand the microscopic origin of thermodynamic changes. Separation of the partition functions into two terms, the Boltzmann factor and the effective volume, allows for a clearer understanding of changes in the partition function as given by Equation (19) (Figure 1). The former is controlled by thermal processes and is thus associated with energy. At equilibrium, a unique temperature rules the frequency probability with which each fragment appears to depend on its energy level shown in Figure 2a. In contrast, in non-equilibrium, the probability should not follow a standard Boltzmann-type formula. This is the reason why it is useful to introduce the concept of distribution of configurational temperatures, which accounts for the deviations from the corresponding equilibrium state. As given by Equations (11) and (12) the general formula is illustrated with the two examples shown Figure 5e and Figure 6e. This formula is thought to be applicable to states even far from equilibrium.

The second term is also important, representing simply the volume of the system for an ideal gas. It suggests that caution is required when some external operation is exerted on the system even if no heat exchange occurs. The most famous example is the so-called Maxwell demon [14]. Without any heat exchange in the system, the demon only modifies the direction of the particles movement. The generally accepted explanation is that all the work exerted by the demon should be taken into account**.** According to the present study, the effective volume within which the particles can move is halved because of the Demon’s operation. This interpretation suggests that not only heat exchange but also change in effective volume should be taken into account when any artificial operation affects the movement of the particles. Otherwise, an entropy loss will occur.

## 7. Conclusions

A theoretical framework applicable to the general case of non-equilibrium states even far from equilibrium has been discussed on the basis of an extended canonical partition function from which thermodynamic variables can be calculated. Although the simple relaxation model discussed in this paper did not allow us to explain the fundamental origin of non-equilibrium states or that of the glass transition, the extended canonical partition function does account for the effects of relaxation and glass-like transitions on thermodynamic properties. In a current study this framework is being extended to real phenomena like the glass transition through the use of energy distributions obtained by the simple classical Einstein model. In more general cases, a more sophisticated energy sampling method by MD simulations such as our previous study [15] will have to be developed.

An important point is the use of “spatial sampling”, which has definite advantages over “ensemble sampling” or “temporal sampling”. The former still suffers from the difficulty raised by the concept of ensemble in non-equilibrium states whereas the latter is not applicable to a system that is constantly changing. In addition, spatial sampling makes it easy to understand the origin of changes in thermodynamic variables, which can then be mostly interpreted at any time in terms of structural disorder. Spatial sampling does have two drawback, however. The first is that it requires to define the proper sampling size in either a spatial or an energy space, which one might do, however, by varying this size to detect when additivity of energy breaks down. When it does so, the energy sampled for each fragment differs from one another. The fact indicates that basic energy components begin to be sampled. The second is that a reasonably high number of particles or fragments must be sampled, so that further study is also required to determine it.

## Figures and Tables

**Figure A1:**
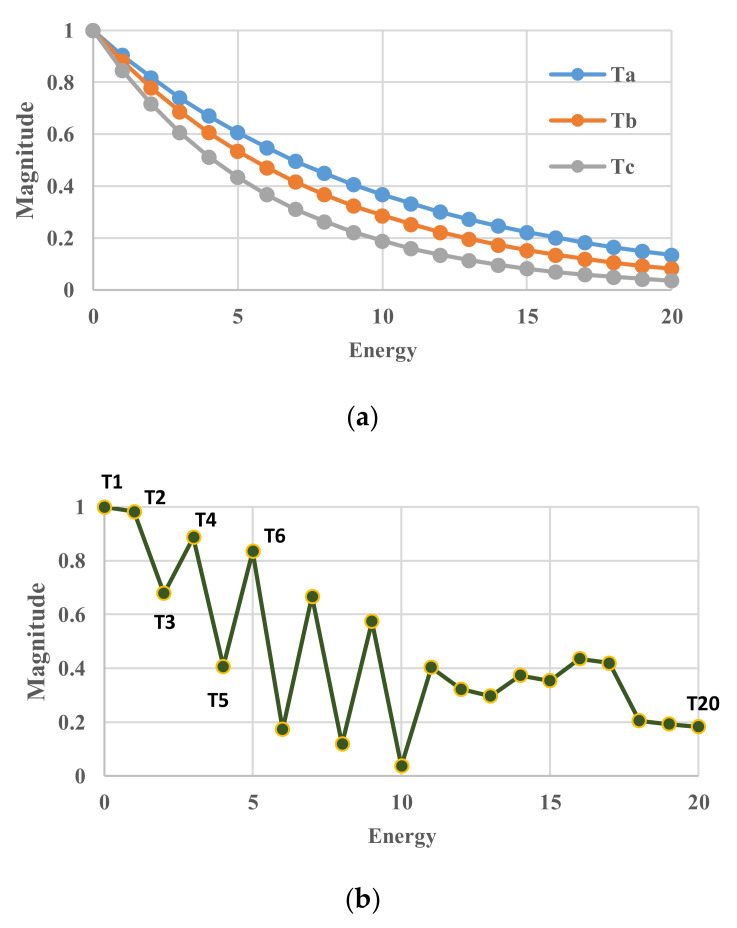
Schematic diagram showing profile of single partition function *g*(*r*) either in equilibrium or extreme non-equilibrium. (**a**) Single partition function *g*(*r*) in equilibrium as a function of energy. Three profiles are the function in equilibrium at different temperature *Ta*, *Tb*, *Tc*, respectively; (**b**) Single partition function *g*(*r*) in extreme non-equilibrium. A single temperature value cannot express the curve of *g*(*r*), however, the introduction of multiple temperature enables it.

**Figure A2:**
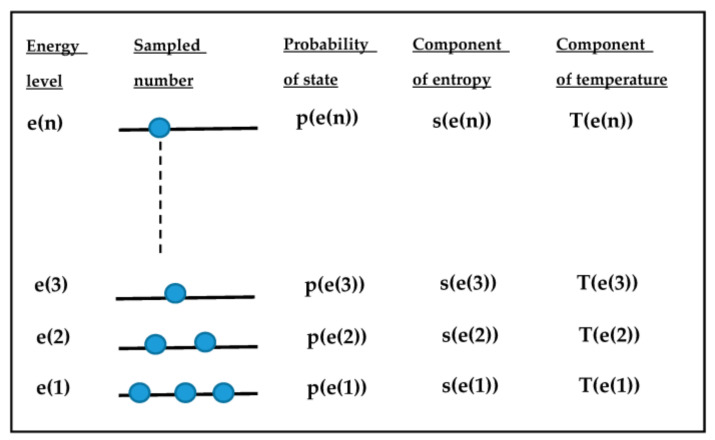
Multi-variable scheme appearing in the new theoretical model.

**Figure A3:**
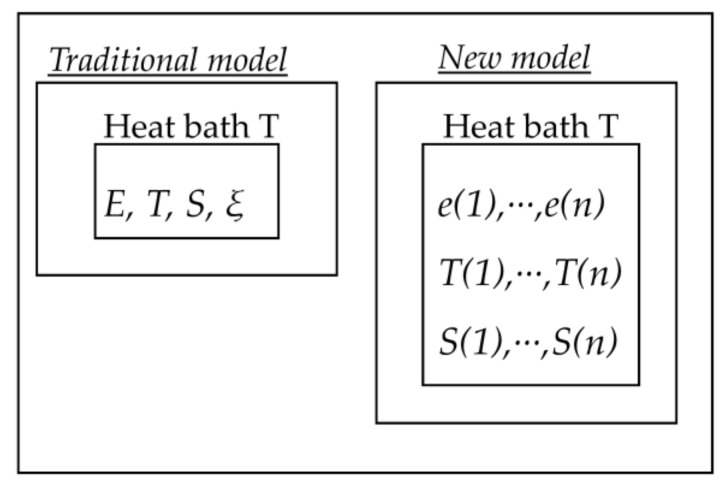
Comparison between traditional and new model. *E* and *e*(*i*) are internal energies, *T* and *T*(*i*) are temperatures, *S* and *S*(*i*) are entropies, *ξ* is a reaction variable as used in affinity approach.

## Appendix A

In this Appendix, we discuss further the application of “spatial sampling” to estimating the partition function further by using the simple example of a one-dimensional classical crystal with a potential *e*(*r*) that is a function of distance from the equilibrium position. The single-particle partition function *z_r_* in equilibrium is analytically expressed as the integration of the components of *p_r_*(*r*):

(A1) $$p_{r}\left(e\left(r\right)\right)=\exp\left(-e\left(r\right)/\left(k_{B}T\right)\right)$$

(A2) $$z_{r}=\int_{0}^{\infty }p_{r}\left(e\left(r\right)\right)dr.$$

By random “spatial sampling”, each sampling of particle unveils its value of energy *e* although the information on distance *r* remains lacking If the histogram of frequency of occurrence of energy *e* is plotted as a function of *e*, it shows polygonal curves approximating the smooth curve of *p_r_*(*e*), however, accompanied by an unknown constant scaling factor on the *y* axis. To get the good estimate of *p_r_*(*e*), there are two important points. The first is that a sufficient number of sampling is required to approximate the curve of *p_r_*(*e*). Statistics tells that the higher number of sampling is taken, the polygonal curve approaches better the smooth theoretical curve of *p_r_*(*e*) after introducing an appropriate scaling factor. The second is to identify the scaling factor that is equivalent to the *d* parameter appearing in Equation (9). When the sampling number is increased *n* times, the height of histogram is also increased *n* times. In the simple one-dimensional case, if the value of lowest energy of *e* is set to be the point of origin (i.e., 0), the value of *p_r_*(*e*) turns to be exp(−0/*k_B_T*), i.e., always to be 1. Therefore, it is easy to scale the *d* parameter so that the value of the extreme left of *p_r_*(*e*) should be 1. On the other hand, in the more general three-dimensional cases, the term of *g_r_*’(*e*) is added in *z_r_* as shown in Equation (20). Hence, the procedure for identifying the *d* parameter is not simple. More studies are required in future.

It is interesting to note that in the infinite temperature limit, Equation (9) leads to *z_s_* = *d N_s_*, which indicates that the partition function *z_s_* is linked to the accessible number of states *Ns* by multiplying *d*. This fact may suggest some physical meaning of the *d* parameter.

## Appendix B

There are several reasons why multiple temperatures named *pseudo-temperature* have to be introduced. The components of single-partition function per particle is defined as the form of Equation (3). As shown in Figure A1a, the profile depends on temperature but it has always the same form of exp[*e*(*i*)/(*k_B_T*)]. The single value of temperature dictates the whole curve. In contrast, if more general situations in non-equilibrium are supposed, any curve is feasibly theoretically. One example of imaginary curves is shown in Figure A1b. The curve cannot be expressed with a single temperature, but with 20 values of temperature. It is proper to say that each value of *pseudo-temperature* does not indicate a real temperature in the conventional sense, but one array of temperatures, i.e., *a vector*, expresses its probability distribution of occurrence of each energy level.

The conventional value of *fictive temperature* can be approximated from the curve of *g*(*r*). In equilibrium, the single value of temperature calculated from the curve of *g*(*r*) may be called *single configurational temperature or fictive temperature* and it is equivalent to the *kinetic temperature* calculated from the velocity distribution of particles. In non-equilibrium, such *single value of configurational temperature* deviates from the *kinetic temperature*. The *pseudo-temperature* or *multiple configurational temperature* is thought to be an extension of the conventional *fictive temperature*, because the *pseudo-temperature* has the same meaning as the *fictive temperature* when all multiple temperatures have the same values.

The meaning of partition function and pseudo-temperature is discussed in further detail from another point of view by using Figure A2. In equilibrium, once the heat bath temperature *T* is fixed, the distribution of sampled number or its normalized value *p*(*e*(*i*)) (i.e., the probability of each state) is uniquely solved. On the other hand, a single temperature cannot distinguish each possible different non-equilibrium state. Historically, the concepts of affinity or fictive temperature have been introduced to allow for this distinction. The introduction of partition function originates from the authors’ idea that the distribution of *p*(*e*(*i*)) can uniquely distinguish any thinkable non-equilibrium state in return for bringing multiple parameters into the new model. Of course, it would be possible to use *p*(*e*(*i*)) instead of *T*(*e*(*i*)); the approaches would be equivalent. However, it seems preferable to use straight-forward thermodynamic variables. In this context, it is interesting to note that both energy and entropy are extensive and internal system variables, whereas temperature is an intensive and, in general, an external system variable. The introduction of *psedo-temperature* enables the new model to define additional temperatures as new internal system variables, which are different from the heat bath temperature. In Figure A3, the approach of the new model is compared with the traditional model based on *affinity*. The merit of the concept of *pseudo-temperature* rests on the possibility that it can be defined under both adiabatic conditions, and under conditions in which the system is in non-equilibrium contact with the heat bath. A further example is the case where irradiation changes structure and energy distribution in an unusual way. It is not possible to trace such changes by a single variable *ξ* as used in the affinity approach, but by a multiple set of parameters only.

In this study, only the configurational part of partition function is discussed. However, since the total partition function is the product of a configurational and a kinetic part, it would also be possible to introduce kinetic pseudo-temperatures. In all cases where the velocity distribution of atoms deviates from the Maxwell-Boltzmann distribution, the introduction of kinetic pseudo-temperature would enable one to define any arbitrary velocity distribution. Finally, in any case, the total entropy is simply the sum of the kinetic and configurational part.
